# Identification of unique genomic signatures in patients with fibromyalgia and chronic pain

**DOI:** 10.1038/s41598-024-53874-8

**Published:** 2024-02-17

**Authors:** Gayatry Mohapatra, Fabien Dachet, Louis J. Coleman, Bruce Gillis, Frederick G. Behm

**Affiliations:** 1 Laboratory of Genomic Medicine, Department of Pathology, University of Illinois at Chicago (UIC) College of Medicine, 840 S. Wood St., Chicago, IL 60612 USA; 2 Department of Medicine, University of Illinois at Chicago (UIC) College of Medicine, Chicago, USA

**Keywords:** Transcriptomics, Medical genomics

## Abstract

Fibromyalgia (FM) is a chronic pain syndrome characterized by widespread pain. The pathophysiology of fibromyalgia is not clearly understood and there are no specific biomarkers available for accurate diagnosis. Here we define genomic signatures using high throughput RNA sequencing on 96 fibromyalgia and 93 control cases. Our findings revealed three major fibromyalgia-associated expression signatures. The first group included 43 patients with a signature enriched for gene expression associated with extracellular matrix and downregulation of RhoGDI signaling pathway. The second group included 30 patients and showed a profound reduction in the expression of inflammatory mediators with an increased expression of genes involved in the CLEAR signaling pathway. These results suggest defective tissue homeostasis associated with the extra-cellular matrix and cellular program that regulates lysosomal biogenesis and participates in macromolecule clearance in fibromyalgia. The third group of 17 FM patients showed overexpression of pathways that control acute inflammation and dysfunction of the global transcriptional process. The result of this study indicates that FM is a heterogeneous and complex disease. Further elucidation of these pathways will lead to the development of accurate diagnostic markers, and effective therapeutic options for fibromyalgia.

## Introduction

Fibromyalgia (FM) is a chronic pain syndrome that for decades has been questioned as a medical disease or merely a collection of symptoms. Those symptoms include chronic, non-remitting pain, body area tenderness, persistent fatigue, recurrent headaches, “brain fog,” generalized anxiety, chronic depression, poor sleep, leg cramps, numbness and tingling, difficulty concentrating and restless legs while sleeping^1^. “Fibrositis” was the first name assigned to this collection of medical complaints. The first proposed criteria for identifying FM patients were put forth in 1990 by the American College of Rheumatology for the purpose of identifying patients for research^2^. The proposed criteria included a history of widespread pain and eighteen designated tender points on physical examination. In 2016, the American College of Rheumatology put forth provisional criteria for FM and revised these in 2016^3–5^. However, these criteria are difficult to assess in the clinical realm and are not widely accepted such as evidenced by another diagnostic system proposed by the American Pain Society in 2019^5^.

While there are no biomarkers available for accurate diagnosis of FM, our group used a multiplex cytokine assay, to report cytokine profiles for FM patients^6^. This study showed that pro-inflammatory cytokines such as IL6, IL8, MIP-1 α (CCL3) and MIP-1 β (CCL4) are consistently under expressed in FM patients as compared to a control group of healthy individuals. Subsequently, a second independent study demonstrated that these biomarkers were unique to FM and did not occur in rheumatoid arthritis or systemic lupus erythematosus^7^.

Current investigations into the pathophysiology of FM have focused on the immune system (e.g., inflammatory and anti-inflammatory cytokines)^1^, the nervous system (e.g., neuro-immune axis, pain processing, neurotransmitters, autonomic nervous system)^8^, the digestive system (a gut-brain axis and the gut microbiome)^9,10^, and genetics (e.g., genome-wide linkage analysis, twin studies, pain and neurotransmitter gene abnormalities)^11–14^. Various approaches have been used to characterize molecular and biological pathways involved in FM. Study investigating thrombosis-related parameters in patients with FM reported elevated platelet, RBC counts and fibrinogen levels and a decreased prothrombin time suggesting an enhanced inflammatory tone and possible risk of thrombosis-related cardiovascular disease^15^. Increased levels of C-reactive protein (CRP) and apolipoprotein B, two biomarkers linked to cardiovascular events in FM has also been reported^16^. Microarray based gene expression analysis has implicated an autoimmune component in the pathogenesis of FM with the presence of dual gene signatures of TH17 and type I interferon; higher levels of IL-17 producing CD4+ T cells and serum cytokines such as TGF-beta, IL-6, IL-21 and IL-23 that promote Th17 differentiation confirming the presence of chronic inflammatory process^17^. Proteomic analyses of plasma proteins from women with FM have shown increased levels of specific plasma proteins suggesting systemic differences in protein expression associated with different clinical parameters^18,19^. A hypomethylated DNA pattern enriched for genes implicated in stress response and DNA repair has also been reported in FM^20^. However, the findings of many of these studies are often contradictory, including a small number of samples and many have not been independently confirmed. Finally, Chronic pain may not always be linked to genes but may be caused through interaction between genes, environmental and life style factors^21^. Due to significant phenotypic heterogeneity, multi-omics approaches may shed light on the underlying biological processes involved in FM and chronic pain.

To gain a comprehensive overview of the transcriptional processes and to define a potential genomic signature of FM, we performed high throughput RNA sequencing (RNA-seq) on peripheral blood mononuclear cells (PBMC) from 96 FM patients and 93 control individuals. Inclusion in the FM group required meeting the criteria of the 2016 College of Rheumatology and having a positive FM cytokine assay^3,6^. Our major objectives were to identify transcriptional differences between FM and healthy controls; to identify subgroups within the FM cohort, and to improve diagnosis and patient stratification using FM specific biomarkers. First, we assessed differentially expressed genes in FM cases compared to healthy controls. We then used bioinformatic tools to identify subgroups of FM patients with distinct genetic signatures.

## Material and methods

### Study participants

This study was performed with the approval of the institutional review board of the University of Illinois at Chicago (Office for the Protection of Research Subjects, OPRS) and all methods were performed in accordance with the relevant guidelines and regulations. The study groups consist of 96 FM patients (91 females and 5 males) and 93 control cases (41 females and 52 males). The participants (FM and controls) included in this study did not overlap with the participants from the previous study published in 2012^6^. All participants provided written informed consent. The inclusion criteria for FM followed the 2016 criteria of the American College of Rheumatology^3–5^ and had a positive fibromyalgia assay (FM/a)^3,6^. The control group did not fulfill the 2016 criteria of the College of Rheumatology and had a negative fibromyalgia assay (FM/a). The FM/a included expression analysis of four cytokines, IL6, IL8, Mip1-α/CCL3 and Mip1-β/CCL4 and relied on the functioning of viable PBMC as previously described^6^. Exclusion criteria, both for patients and controls, were the presence of any other chronic disease (diabetes, heart disease or cancer). A questionnaire was used for the collection of demographic and clinical data from participants. None of the patients were treated with anti-inflammatory drugs at the time and 3 months before the start of the study.

### Sample collection, RNA extraction, library construction and sequencing

For genomic analyses, blood samples (9–10 mL) from FM and control individuals were collected in Streck tubes (Streck, La Vista, NE). Samples were centrifuged at 2500 rpm to separate the plasma, PBMC and RBC layers. After removing plasma, the RBCs were lysed using Qiagen RBC lysis buffer and centrifuged for 20 min at 2500 rpm at room temperature. Cell pellets were washed in PBS twice to remove any trace of RBC, then homogenized using a QIAshredder homogenizer in 600 μL RLT buffer containing β-mercaptoethanol. Total RNA was extracted using QIAamp RNA blood mini kit (Qiagen, Germantown, MD). RNA quality was assessed using Agilent RNA screen tape and TapeStation 4200 (Agilent Technologies, Santa Clara, CA) and a nanodrop spectrophotometer was used to estimate the concentration and purity of RNA (Nano-Drop Technologies, Wilmington, DE).

RNA libraries were prepared using Agilent SureSelectXT RNA Direct workflow following manufacturer’s instructions. Briefly, 200 ng of RNA was transferred into strip tubes and samples were completely dried at 30 °C in a vacufuge (Eppendorf). RNA-seq fragmentation mix was added to each sample, mixed by vortexing gently at 2000 rpm for 10 s. Fragmentation was carried out at 94 °C for 8 min and kept at 4 °C. RNA-seq first strand master mix was added to each sample and the first strand was synthesized using the following thermal cycling condition 25 °C (10 min), 37 °C (40 min) then maintained at 4 °C with heated lid on. The first stand was purified using Agencourt AMPure XP beads (Beckman Coulter Genomics). Second-strand cDNA was synthesized and end-repaired at 16 °C for 1 h. The second-strand cDNA was purified using Agencourt AMPure beads. The 3' ends of cDNA were dA-tailed at 37 °C for 30 min and adaptors were ligated to each dA-tailed cDNA at 20 °C for 15 min. The adaptor ligated cDNA was purified using Agencourt AMPure beads and was amplified using pre-capture thermal cycling conditions. The quality of the pre-captured library was assessed using D1000 ScreenTape on Agilent 4200 TapeStation system. The region between 150 and 400 bp was used for quantification. Hybridization was carried out overnight at 65 °C in a thermal cycler using 200 ng of pre-captured library. For capturing the targets, SureSelectXT Human All Exon V6 baits were used (Agilent Technologies Inc., Santa Clara, CA). The captured libraries were then amplified to add the index tags and were purified using Agencourt AMPure beads and finally eluted in low TE buffer. The quality and quantity (region of 150–500 bp) of libraries were assessed using high sensitivity D1000 screen tape on a 4200 TapeStation system. Paired-end sequencing was performed at 2 ng/µL concentration on a NovaSeq 6000 system pooling 25 libraries/S4 flow cell (Illumina, San Diego, CA) with an average of 136 million reads per sample. Raw data have been submitted to NCBI and GSE221921 provides access to all data (https://www.ncbi.nlm.nih.gov/gds/?term=GSE221921).

### Data analysis

FASTQ files corresponding to the forward and reverse reads for 189 samples in total, 96 FM and 93 control were obtained from Illumina BaseSpace and used for analysis. The files were processed using the Trim Galore (Babraham Bioinformatics) and Cutadapt (DOI: 10.14806/ej.17.1.200) tools to perform a quality trimming by removing short, low-quality reads and the adapters. RNA-seq reads were mapped to the reference genome (Gencode.v38) and aligned using STAR aligner^22^. Duplicate reads were removed, and uniquely mapped transcripts were selected using Samtools^23^. The TPMs (Transcripts per million) were computed using StringTie^24^ corrected by IsoformSwitchAnalyzeR^25^ and normalized using TMM (Trimmed Means of M value)^26^. Differential expression analysis was performed using DESeq2^27^, using all annotated genes. Ontological analysis of gene expression was carried out using the Qiagen Ingenuity Pathway Analysis (IPA), and Gene Set Enrichment Analysis (GSEA)^28^. The Interactome analysis was carried out by Pearson Correlation clustering using the 914 most differentially expressed genes for FM1, the 361 most differentially expressed genes for FM2 and the 402 most differentially expressed genes for FM3 and FM4 using Cytoscape^29^, the clusters were determined by AlegroMcode (AllegroViva Corporation, 2011) using default parameters.

## Results

### Clinical characteristics of FM patients

The median age of patients participating in this study was 48 years of age with overall ages ranging from 28 to 77 years. The median age of the onset of FM was 36 years of age. The median age of control patients was 39 years, with ages ranging from 20 to 69 years. The clinical characteristics of the entire cohort are presented in Table 1.

**Table 1:** Clinical characteristics of FM and control groups.

Clinical features	FM group (96)	Control group (93)
Gender	F- 91, M–5	F–43, M–50
Age	Median–48 (range 28–77)	Median–45 (range 20–69)
Age of onset/diagnosis	Median–36 (range 12–66)	N/A
Muscle/Body pain	93 (97%)	0
Tender areas	91 (95%)	0
Chronic fatigue	92 (96%)	4 (4%)
Sleep disorder	85 (88.5%)	4 (4%)
Anxiety	77 (80%)	11 (12%)
Joint aches	84 (87.5%)	6 (6.5%)
Frequent headaches	56 (58%)	2 (2%)
Restless legs/Leg cramps	69 (72%)	5 (5.4%)
Numbness or tingling	74 (77%)	6 (6.5%)
Trouble remembering	87 (90.6%)	0
Trouble concentrating	90 (94%)	8 (8.6%)
Depression	64 (67%)	10 (10.8%)

**Figure 1:**
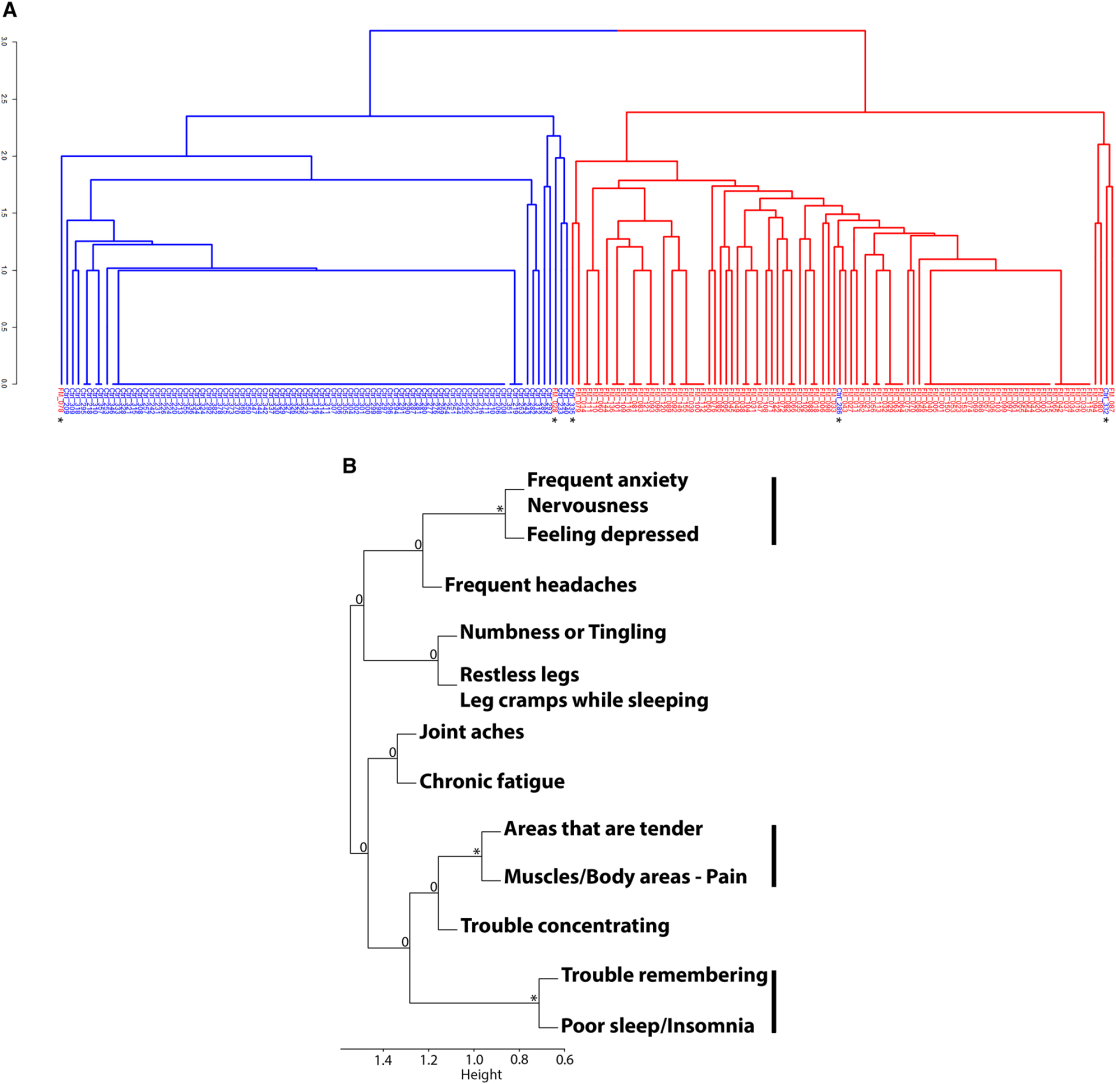
Hierarchical clustering of FM patients and controls. (**A**) Hierarchical clustering of patient symptoms indicates a near perfect separation into two groups. A minority of patients were misclassified: three control subjects #286, #332, #335 were assigned to the FM group while two FM patients #028, #078 were assigned to the control group. Average linkage using Euclidean metrics with k = 2 classes, control patients represented in blue, FM patients represented in red, misclassified patients are identified by an asterisk. (**B**) Clustering of FM patient symptoms. The symptoms were grouped by hierarchical clustering using average linking of Pearson correlation metric. The vertical bars represent the three groups of correlated symptoms that are present. The star '*' or '0' represents the significance of the cluster. '0': No significant correlation, '*' : adjusted *p*-value ≤ 0.01.

We performed hierarchical clustering to test whether using clinical characteristics alone, the FM and control cases can be sub-grouped. A near perfect separation of two groups was observed (Fig. 1A) based on clinical symptoms. Only a minority of cases were misclassified: three control subjects: #286, #332, #335 were assigned to the FM group while two FM patients: #028, #078 were assigned to the control group. These five cases were not included in the downstream analysis.

Hierarchical clustering of patient symptoms in the FM cohort indicated that there were three questions that were redundant: (1) patients with poor sleep and insomnia also had memory impairment, (2) depression was related to anxiety and nervousness, and (3) patients who had body pain were more susceptible to have tender points (Fig. 1B). Principal component analysis (PCA) of the symptoms in FM patients indicate that the non-random explanation of variance is represented only on the first component and accounts for 18% of the observed variance (Supplementary Fig. 1). This suggests that globally the symptoms of FM patients were homogeneous, except for patient #078 who appeared to be the only outlier marked by the absence of body pain and tender areas but instead reported the presence of depression with physical fatigue.

### Cluster analysis of RNA-seq data

**Figure 2:**
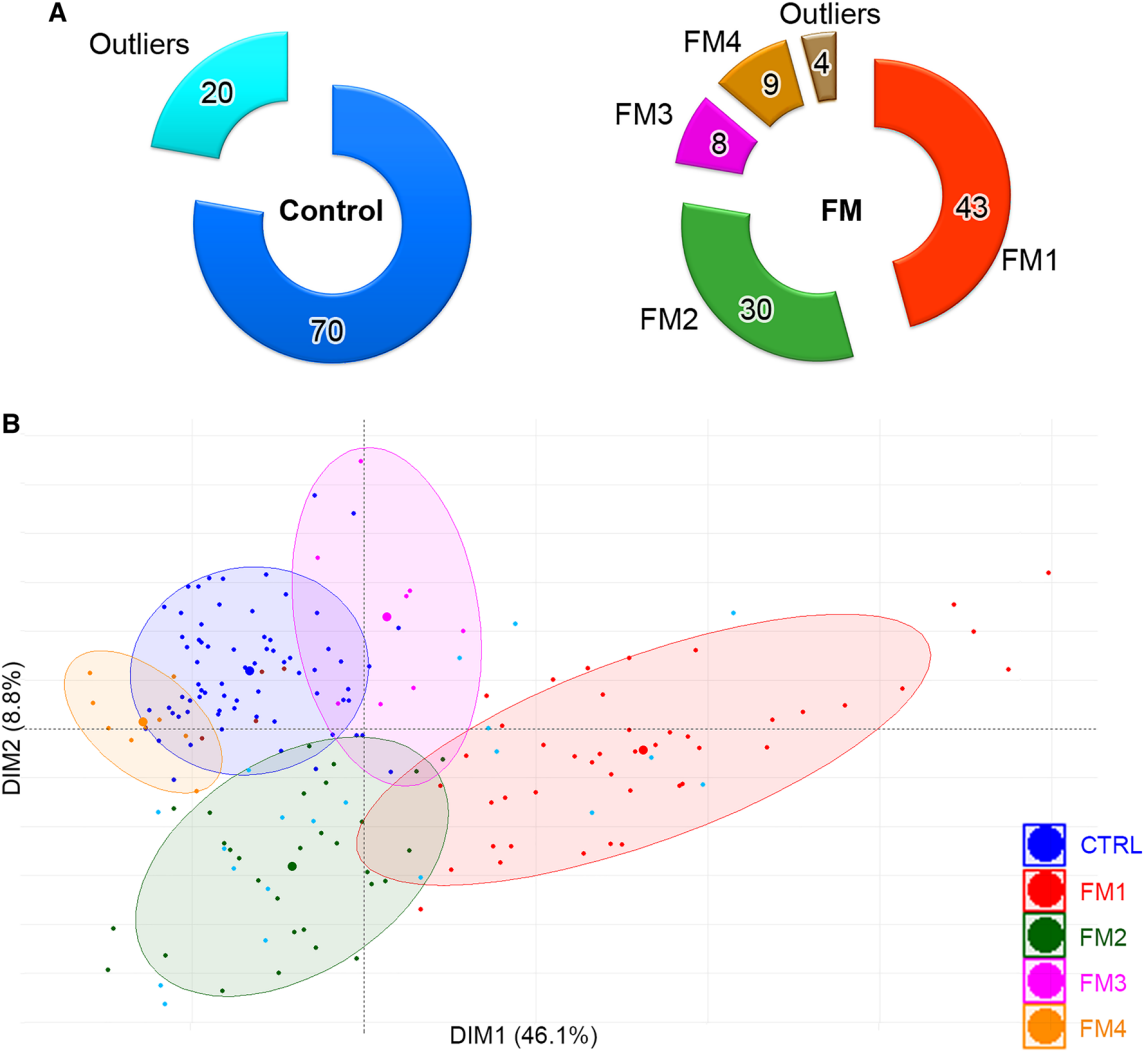
Summary of DEGs identified in FM subgroups. (**A**) Representation of the proportion of controls and FM patients based on their gene expression. 70 control patients with similar gene expression clustered together. The 20 controls that did not have similar gene expression profile are indicated as outliers. Among the FM patients, subgroups with different gene expression profiles were detected. The major group called ‘FM1’ was formed by 43 patients, a second group called ‘FM2’ was formed by 30 patients. The rest of the FM patients could be separated into two clusters of 8 and 9 cases respectively and 4 outliers. (**B**) PCA of FM and controls. PCA of the entire cohort of 94 FM and 90 controls was performed using 1169 DEGs that showed the most significant differential expression. The first component axis shows 46.1% while the second component shows 8.8% of the information. No other components than the first and second components were found useful. Ellipses show 80% confidence interval of each group, the supersized dot corresponds to the centroid of the group. Control patients are represented by a dark blue dot, control patients classified as outliers are represented by a cyan dot, fibromyalgia patients FM1, FM2, FM3, FM4 are represented by a red, green, magenta, orange dot respectively. The 4 FM patients that did not group together are represented by brown dots.

Differentially expressed genes were obtained using linear model with the software ‘R’ (https://www.R-project.org/). To visualize the results of unsupervised clustering we plotted the logarithm of the TPMs using the heatmap function of ‘R’. A total of 1720 differentially expressed transcripts were used to draw the heatmap using the algorithm of TPMs. Of the 90 control cases, 70 formed a tight cluster and 20 were outliers (Fig. 2A, Supplementary Fig. 2). The 20 outliers from the control group dispersed among FM1-3 patients. However, the entire cohort of 94 FM and 90 controls were used for all downstream analyses. The PCA indicated a homogeneous group of 43 patients which we labeled as FM1 and another group of 30 patients labeled as FM2 with unrelated underlying gene expression. The remaining group of 21 patients could be separated into two clusters of 8 and 9 patients each (FM3 and FM4) and 4 outliers (Figs. 2B, 6A). For further analysis, we focused on FM1, FM2 and combined FM3 and FM4 as a group.

A total of 1720 differentially expressed genes (DEGs) were identified in the entire FM cohort with FM1 having 914 DEGs, FM2 having 361 DEGs, FM3 and FM4 having 402 DEGS. Of the 1720 DEGs detected, 1695 were protein coding, 24 lncRNA and 1 miRNA (Supplementary Table 1).

### Analysis of FM1 subgroup

**Figure 3:**
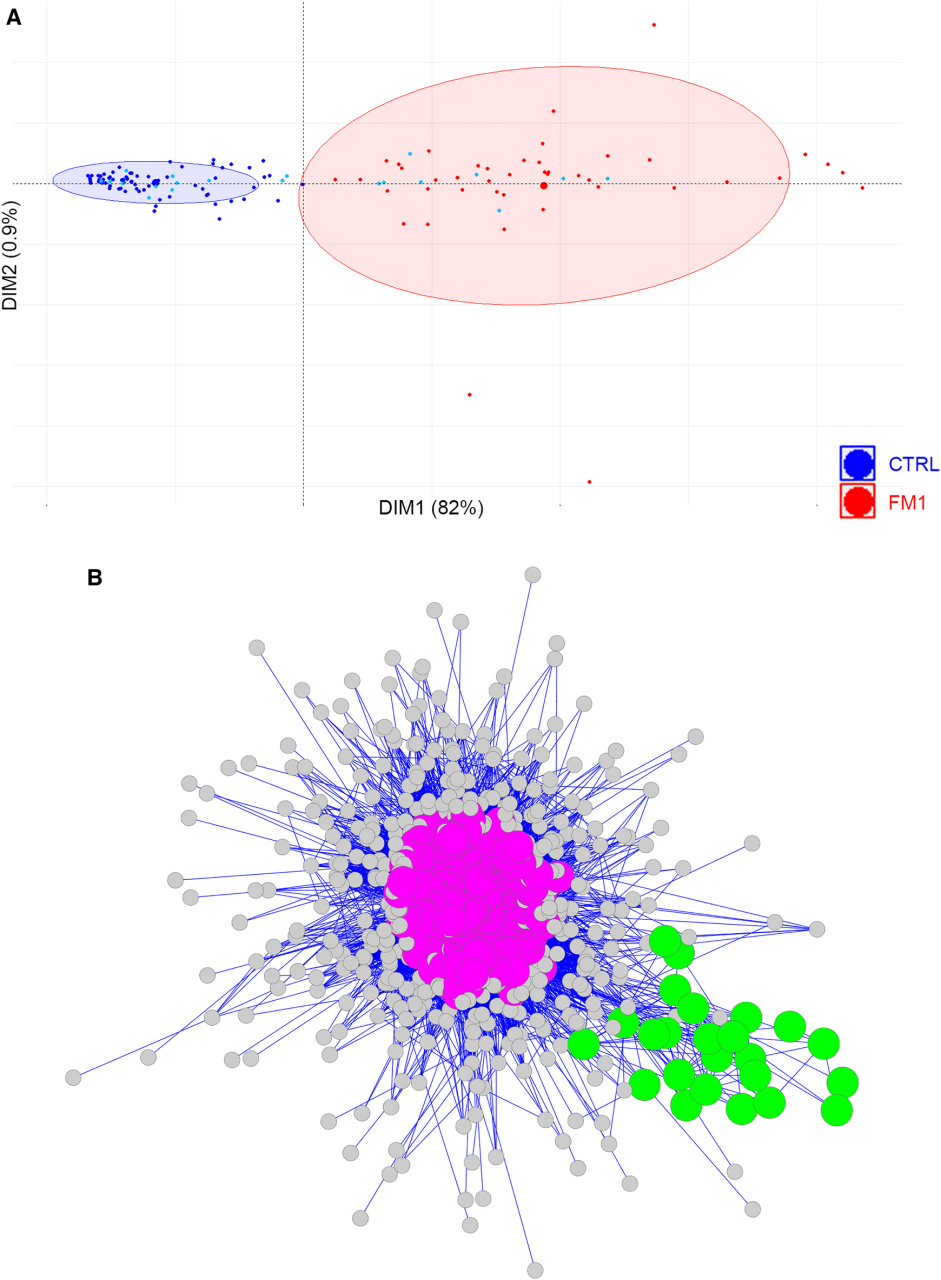

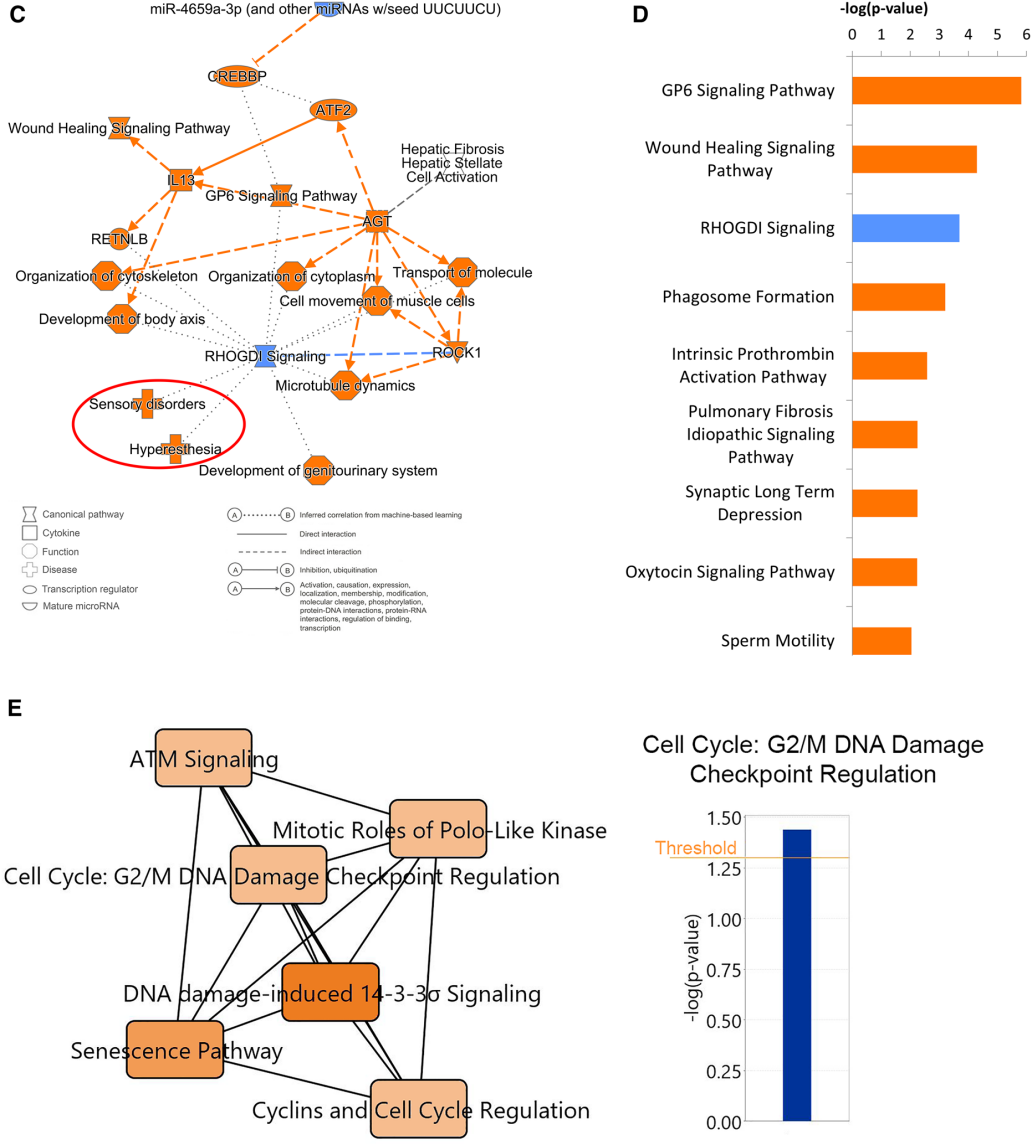
Analysis of DEGs in FM1 subgroup. (**A**) PCA of the 90 controls and the 43 patients of FM1 subgroup using 480 DEGs that showed the most significant differential expression. The near perfect separation shows that most of the variation is represented by the first component (82%). Blue dots: control patients, Red dots: FM patients, the ellipses correspond to the threshold at 80% confidence. Cyan dots show the outlier controls. (**B**) The interactome analysis of 43 FM1 patients showed a group of 338 DEGs (magenta color) and a small cluster of 24 DEGs (green color). Each dot represents a DEG, the gray dots represent DEGs that did not reach significance. (**C**) Pathway analysis of the DEGs in the major cluster (magenta) and the minor cluster (green) using a threshold of –log (*p* value) ≥ 2. (**D**) Summary of biological functions related to the pathways identified in FM1 patients. (**E**) Small clusters of 24 genes (green) including coding and lncRNA from the interactome analysis, are associated with cell cycle regulation. Blue: Downregulation, Orange: Upregulation.

The FM1 subgroup consisted of 43 patients with a similar gene expression pattern. PCA analysis of 480 most DEGs in the 43 FM1 patients and 90 control patients showed a clear separation of patients between FM1 and controls (Fig. 3A). The first component of the PCA encompassed 82% of the total variation indicating that the disease state (Control vs FM1) is the major cause of gene expression difference between these two groups. To understand biological pathways that are specific to the group of 43 FM1 patients, we first performed interactome analysis to identify functional interactions and to pinpoint the DEGs which are most susceptible to being expressed in the same cells. Then we performed IPA on these DEGs to identify the pathways that are associated with these genes. The interactome analysis identified a major cluster composed of 338 DEGs represented in magenta and a smaller cluster of 24 DEGs represented in green (Fig. 3B). The DEGs represented in magenta indicate the presence of a cell (or group of cells) with coordinated gene expression across patients of the FM1 subgroup while the DEGs represented in green appear to represent a biological process. We used the genes included in each of these clusters for IPA analysis and found that the significant pathways represented by the major magenta DEGs belonged to extra-cellular matrix genes involved in connective tissue disorders (pulmonary fibrosis, wound healing, cytoskeletal organization, etc.) (Fig. 3C, Table 2). Also, these DEGs pinpoint the presence of upregulated GP6 pathway and the downregulation of Rho GDP-Dissociation Inhibitors (RHODGI) signaling (Fig. 3D). The minor cluster is composed of 24 DEGs that correspond to cell cycle associated genes (Fig. 3E).

**Table 2:** Top ranked canonical pathways in FM1.

Ingenuity canonical pathways	− log(*p*-value)	zScore	Genes
GP6 signaling pathway	5.82	3.32	COL12A1, COL1A2, COL24A1, COL3A1, COL6A6, COL9A1, FGG, LAMA1, LAMA2, LAMA3, PIK3CA
Wound healing signaling pathway	4.29	1.94	COL12A1, COL1A2, COL24A1, COL3A1, COL6A6, COL9A1, FN1, IL1RAPL2, LAMA1, LAMA2, LAMA3, MMP10, TRAP1
RHOGDI signaling	3.69	− 2.24	CDH10, CDH12, CDH6, CDH8, CDH9, GRIP1, ITGB6, MYH1, MYH2, MYH4, MYH7
Phagosome formation	3.2	4.58	ADGRA3, ADGRB3, ADGRG6, FN1, GPR156, GPRC6A, GRM1, HTR4, ITGB6, LGR5, MYH1, MYH2, MYH4, MYH7, OPRM1, PIK3CA, PLA2G4F, PLA2R1, RAPGEF4, RXFP2, TACR3
Intrinsic prothrombin activation pathway	2.58	2.00	COL1A2, COL3A1, F11, FGG
Pulmonary fibrosis idiopathic signaling pathway	2.24	3.32	COL12A1, COL1A2, COL24A1, COL3A1, COL6A6, COL9A1, FN1, ITGB6, MMP10, MMP20, PIK3CA
Synaptic long term depression	2.24	2.83	GRID2, GRM1, GUCY2C, PLA2G4F, PLA2R1, PLCH1, PRKG2, RYR3
Oxytocin signaling pathway	2.23	2.53	ABCC9, GUCY2C, KCNT2, MYH1, MYH2, MYH4, MYH7, PIK3CA, PLA2G4F, PRKG2
Sperm motility	2.04	2.24	ALK, EPHA3, PDE1C, PLA2G4F, PLA2R1, PLCH1, PRKG2, ROS1, TEK
Cell cycle: G2/M DNA damage checkpoint regulation	1.44	n/a	AC005578.3, CAB39L, CCNB2, CDKN3, CETN3, DNAJC27, EARS2, ELP4, FAM83D, FBXO24, ING1, LINC00167, LYPD3, PPP5D1, RPA2, SARNP, SCOC, SPATA24, SUCO, SYP

### Gene set enrichment analysis (GSEA) of FM1 subgroup

**Figure 4:**
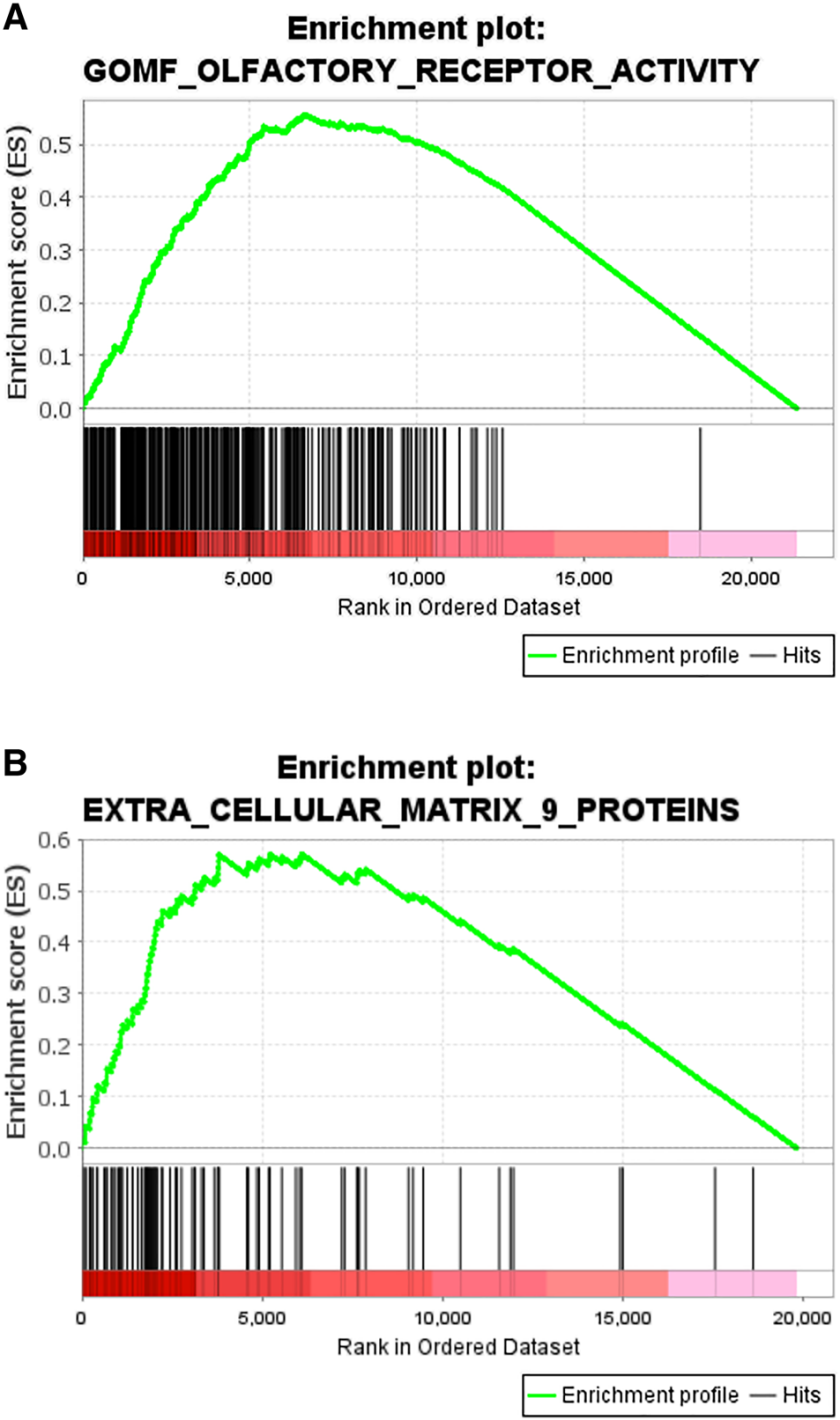
Results of GSEA analysis of the whole transcriptome for FM1 group. (**A**) Enrichment of olfactory receptor activity (Normalized Enrichment Score = 2.1, *p*-value < 0.05). (**B**) Enrichment of the genes expressing nine proteins associated with the extra cellular matrix (Normalized Enrichment Score = 2.0, *p*-value < 1E-4).

Independent of IPA results that searched pathways inside a private database, we also analyzed the whole gene set using GSEA (Gene Set Enrichment Analysis)^28^. This type of analysis ranks the genes from the most differentially expressed to the least differentially expressed and searched among all the known gene sets present in public databases to identify the most enriched gene set. We also analyzed a custom gene set that code for nine proteins composed of the extra-cellular matrix extracted from (Reactome.org): Collagen, Fibrinogen, Elastin, Fibrillin, Fibronectin, Fibulin, Laminin, Matrilin and Tenascin. GSEA of the FM1 dataset revealed that the expression pattern most prominently correlates with olfactory receptor activity (NES = 2.1, *p*-value < 0.05) and extracellular matrix 9 proteins (NES = 2.0, *p*-value < 0.0001) (Fig. 4A,B).

### Analysis of FM2 subgroup

**Figure 5:**
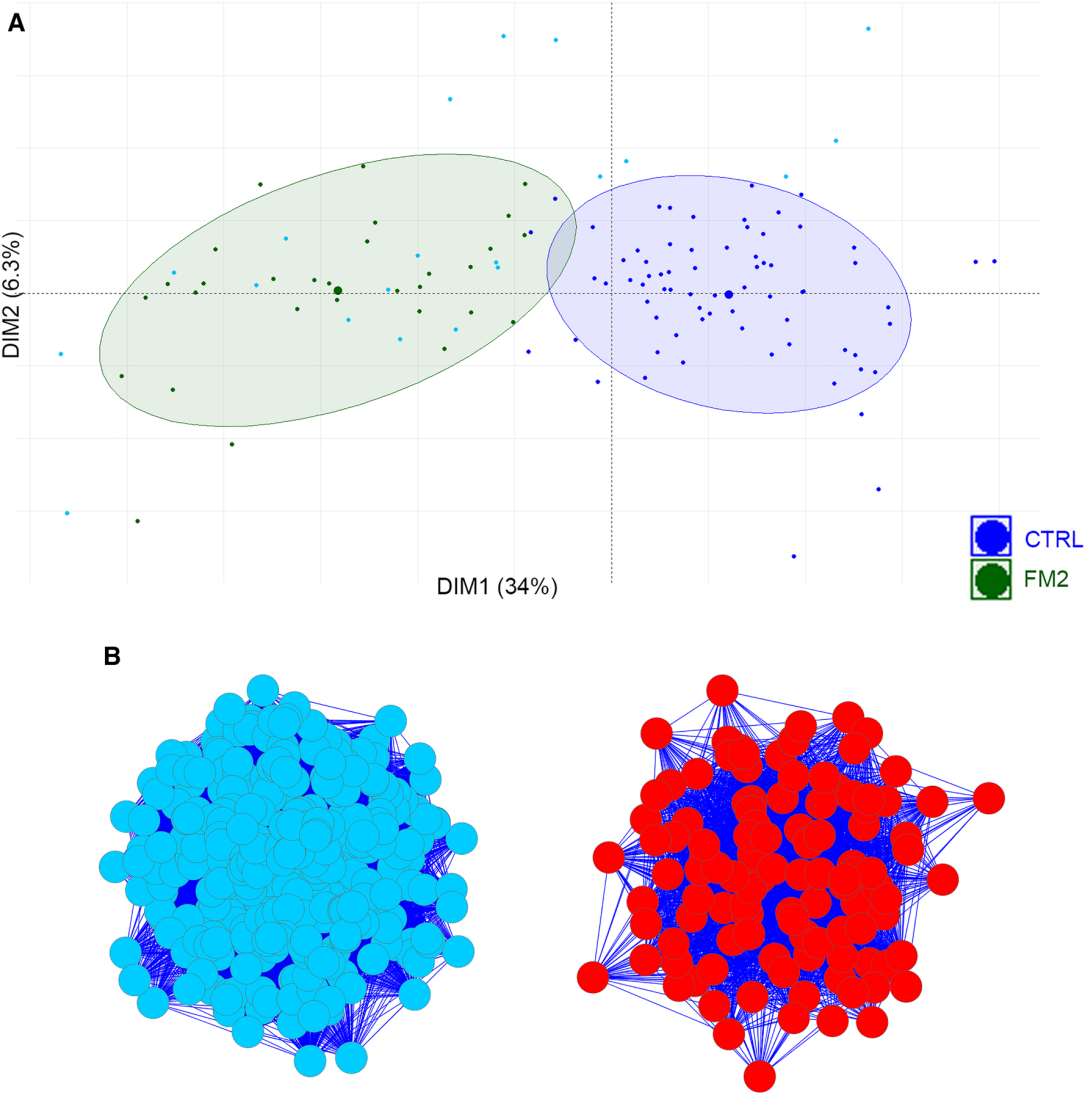

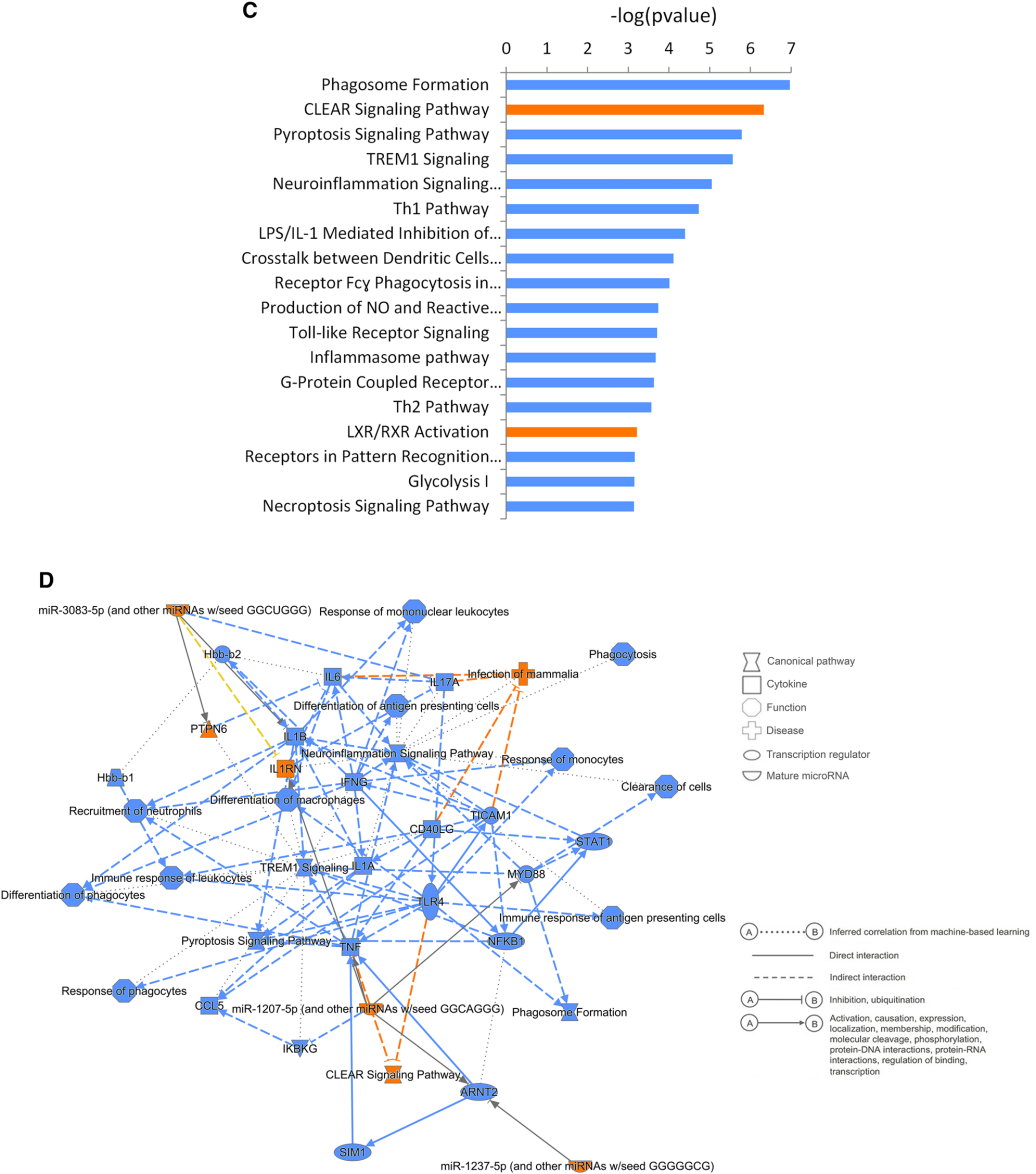
PCA and pathway analysis of FM2 subgroup. (**A**) PCA of the 90 control and the 30 FM2 patients using 361 DEGs. PCA could separate most of the control and FM2 patients. Blue dots: control patients, green dots: FM2 patients, the ellipses correspond to the threshold at 80% confidence. Cyan dots indicate the outlier controls. (**B**) Interactome analysis of 361 DEGs that separated the FM2 using the 90 control patients as reference into two distinct clusters of up and down regulated DEGs. Blue: down regulated genes, Red: up regulated genes. (**C**) IPA analysis shows the most significant pathways found in FM2 until –log (pvalue) ≥ 3. The gene expression indicates numerous pathways associated with inflammatory response are downregulated and the CLEAR signaling pathway is upregulated. (**D**) Summary of biological functions related to the pathways identified in FM2 patients. Blue: downregulation, Orange: upregulation.

The FM2 subgroup was composed of 30 patients who showed a similar type of gene expression pattern. Principal component analysis of the 361 DEGs in the 90 control and 30 FM2 patients showed a separation of patients between the controls and FM2 on the first component (Fig. 5A). The first component of the PCA encompassed 34% of the total variation while the second encompassed 6.3% indicating that the disease state (Control vs FM2) is an important cause of gene expression differences between these two groups of patients. Interactome analysis separated the DEGs of the FM2 patients into two distinct clusters (Fig. 5B). These 2 clusters correspond to the up-regulated and down-regulated DEGs. IPA analysis of the DEGs identified that the most significant results in this group were the suppression or dysregulation of inflammatory processes (Fig. 5C). The top-ranked dysregulated pathways include phagosome formation, pyroptosis signaling pathway, TREM1 signaling, neuro-inflammation signaling, Th1 pathway, IL1-mediated inhibition of RXR function, crosstalk between dendritic cells and natural killer cells, toll-like receptor signaling, inflammaosome pathway, Th2 pathway (Fig. 5D, Table 3). These results indicate a lymphocyte to monocyte ratio imbalance in FM2 patients (Fig. 5C). The CLEAR signaling pathway and LXR/RXR activation pathways were upregulated (Fig. 5D).

**Table 3:** Top ranked canonical pathways in FM2.

Ingenuity canonical pathways	− log(*p*-value)	zScore	Genes
Phagosome formation	6.97 removing to present data consistently from table 2–4	− 4.85	ADGRE1, ADGRE3, ADGRG1, AP1S2, C5AR1, CCR1, CCR2, CD14, CD36, FCER1G, FCGR1A, FCGR2A, FGR, FPR1, FPR2, HCK, HMOX1, HRH2, IGHM, ITGAM, LPAR1, LYN, P2RY13, PAK1, PLB1, PRKCD, PTAFR, S1PR3, TLR2, TLR4, TLR8
CLEAR signaling pathway	6.33	0.47	ASAH1, ATP6V0B, ATP6V0C, ATP6V0D1, ATP6V1B2, DDIT4, GAA, GABARAP, **GNS**, IFI30, INSR, PRKCD, **PSAP**, RXRA, TLR2, TLR4, TLR8, TNFRSF1B
Pyroptosis signaling pathway	5.79	− 3.16	CASP4, IL1B, MEFV, NLRC4, NLRP12, PYCARD, TLR2, TLR4, TLR8, TNFRSF1B
TREM1 signaling	5.57	− 3.00	CD86, IL1B, NLRC4, NLRP12, TLR2, TLR4, TLR8, TREM1, TYROBP
Neuroinflammation signaling pathway	5.05	− 3.36	CD86, CSF1R, CYBB, FOS, GABRR3, GRINA, HMOX1, IFNA4, IFNGR2, IL1B, NCF2, PSEN1, PYCARD, TLR2, TLR4, TLR8, TREM1, TYROBP
Th1 pathway	4.73	− 0.71	CD247, CD86, GATA3, IFNGR2, KLRD1, LGALS9, NOTCH2, PSEN1, STAT4, TBX21
LPS/IL-1 mediated inhibition of RXR function	4.39	− 1.34	ALDH2, ALDH3B1, CD14, CHST15, CYP2S1, GSTP1, IL1B, IL1RN, NDST1, RARA, RXRA, SULT1A1, TLR4, TNFRSF1B
Crosstalk between dendritic cells and natural killer cells	4.11	− 0.71	CD226, CD86, KLRD1, LTBR, PRF1, TLR4, TNFRSF1B, TYROBP
Fcγ receptor-mediated phagocytosis in macrophages and monocytes	4.01	− 2.83	FCGR1A, FCGR2A, FGR, HCK, HMOX1, LYN, PAK1, PRKCD
Production of nitric oxide and reactive oxygen species in macrophages	3.74	− 3.16	CYBB, FOS, IFNGR2, LYZ, NCF2, PRKCD, SIRPA, SPI1, TLR2, TLR4, TNFRSF1B
Toll-like receptor signaling	3.71	− 2.24	CD14, FOS, IL1B, IL1RN, TLR2, TLR4, TLR8
Inflammasome pathway	3.67	− 2.00	IL1B, NLRC4, PYCARD, TLR4
G-protein coupled receptor signaling	3.63	− 2.86	ADGRE1, ADGRE3, ADGRG1, AMOT, ARRB2, C5AR1, CCR1, CCR2, DUSP1, DUSP6, FOS, FPR1
Th2 pathway	3.57	− 1.00	CCR1, CD247, CD86, GATA3, NOTCH2, PSEN1, SPI1, STAT4, TBX21
LXR/RXR activation	3.21	1.41	CD14, CD36, IL1B, IL1RN, LYZ, RXTA, TLR4, TNFRSF1B
Role of pattern recognition receptors in recognition of bacteria and viruses	3.16	− 1.89	C5AR1, IFNA4, IL1B, NLRC4, OAS1, PRKCD, TLR2, TLR4, TLR8
Glycolysis I	3.15	− 2.00	ALDOA, FBP1, GAPDH, PKM
Necroptosis signaling pathway	3.14	− 1.67	CASP10, CYBB, IFNA4, PELI1, PYCARD, PYGL, SLC25A5, TLR4, TNFRSF1B

### Analysis of FM3 and FM4 subgroups

**Figure 6:**
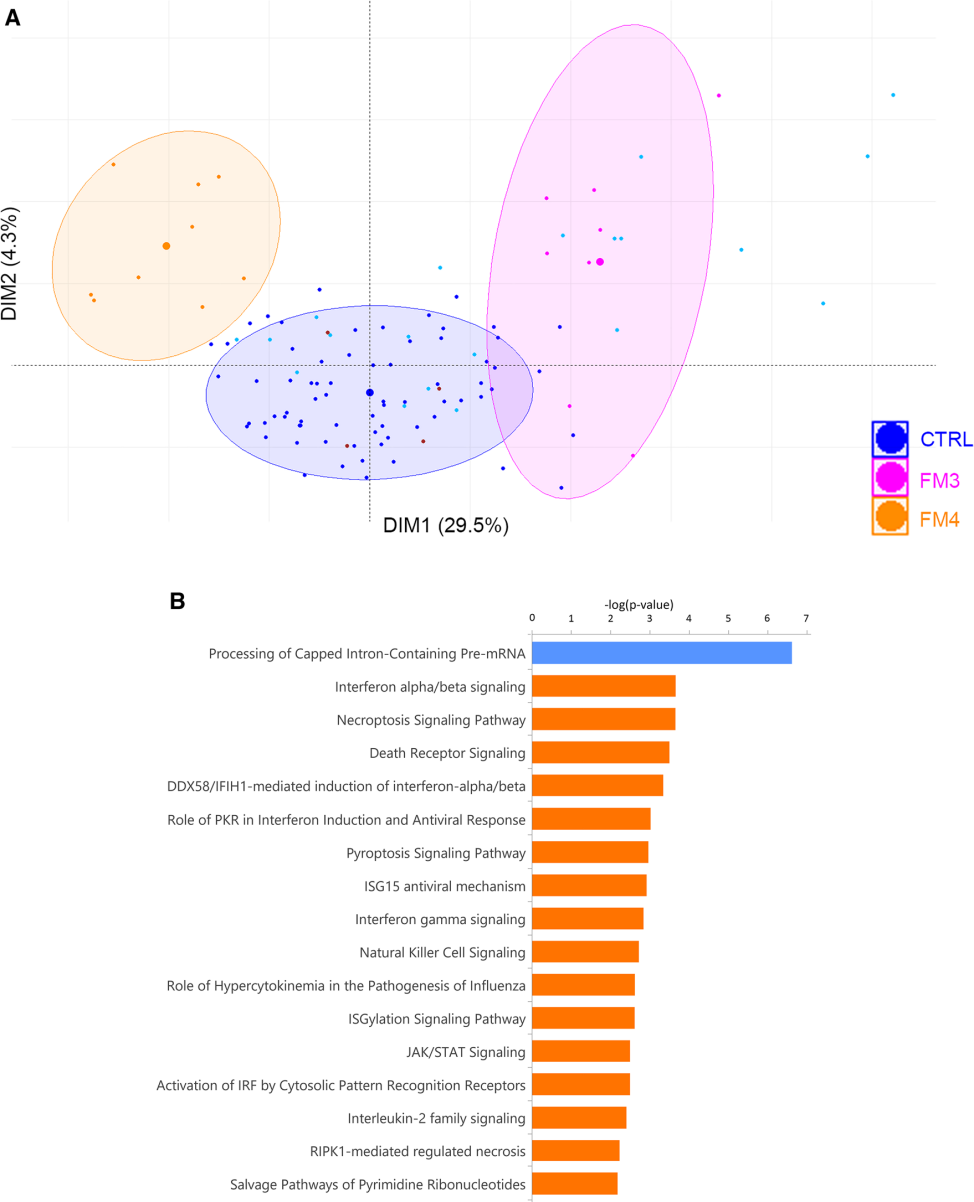
PCA and pathway analysis of FM3 and FM4 subgroups. (**A**) First component axis is showing 29.5% and was used for separation of control patients from FM3 and FM4. Ellipses show 80% confidence interval of each group, and the supersized dots correspond to the centroid of the group. Control patients are represented by a dark blue dot, control outliers are represented by a cyan dot, FM3 are represented by a magenta dot while FM 4 are represented by an orange dot. The 4 outlier FM cases were represented by brown dots. (**B**) IPA analysis shows the most significant pathways found in FM3&4 until –log (pvalue) ≥ 2. The gene expression indicates numerous pathways associated with acute inflammatory processes are upregulated and the processing of pre-mRNA pathway is downregulated.

The FM3 and FM4 subgroups were composed of 17 cases together. Principal component analysis of the 361 DEGs in the 90 control and 17 FM patients showed a separation of patients between the controls, FM3 and FM4 on the first component (Fig. 6A). The first component of the PCA encompassed 29.5% of the total variation while the second encompassed 4.3% indicating that the disease state (Control vs FM3 and 4) is an important cause of gene expression differences between these groups of patients. Interactome analysis did not establish any significant interactions due to the small sample size. IPA analysis of the DEGs identified the following top-ranked dysregulated pathways including interferon signaling, death receptor signaling, natural killer cell signaling, JAK/STAT signaling and the processing of capped intron-containing pre-mRNA pathway (Fig. 6B, Table 4).

**Table 4:** Top ranked canonical pathways in FM3 and FM4.

Ingenuity canonical pathways	−log(*p*-value)	zScore	Genes
Processing of capped intron-containing pre-mRNA	6.62	− 0.63	ACIN1, BUD13, CCAR1, CSTF2, CWF19L2, FYTTD1, HNRNPA3, MAGOH, MAGOHB, METTL14, MTREX, NCBP1, PCF11, PRPF3, PRPF38A, PRPF4, PRPF40A, RBM39, RBM7, SRSF4, WDR70, WTAP, ZMAT2
Interferon alpha/beta signaling	3.66	2.12	ADAR, ISG20, MX2, PTPN6, SAMHD1, STAT1, STAT2, XAF1
Necroptosis signaling pathway	3.65	1.73	CASP1, CASP10, CFLAR, EIF2AK2, IKBKB, MLKL, PPP3CB, STAT1, STAT2, TIMM13, TNFSF10, ZBP1
Death receptor signaling	3.50	1.00	ACIN1, CASP10, CSFLR, IKBKB, MAP4K4, PARP2, PARP4, PARP9, TNFSF10
DDX58/IFIH1-mediated induction of interferon-alpha/beta	3.34	0.71	CASP10, HERC5, HSP90AA1, IKBKB, NLRX1, RIG1, TAX1BP1, TRIM25
Role of PKR in interferon induction and antiviral response	3.02	1.27	CASP1, EIF2AK2, HSP90AA1, HSPA5, IKBKB, METAP2, RIGI, STAT1, STAT2, TIRAP
Pyroptosis signaling pathway	2.96	2.12	CASP1, GBP1, GBP4, GBP5, MEFV, PRKAR1A, PTGER4, TXNIP
ISG15 antiviral mechanism	2.92	1.13	EIF2AK2, HERC5, KPNA2, MX2, RIG1, STAT1, TRIM25
Interferon gamma signaling	2.84	2.12	GBP1, GBP4, GBP5, PTPN6, STAT1, TRIM22, TRIM25, TRIM38
Natural killer cell signaling	2.72	1.16	CFL1, HSPA5, LAT, MICA, NFAT5, PIK3C3, PIK3R1, PTK2B, PTPN6, RAP1B, STAT4, TNFSF10
Role of hyperchemokinemia in the pathogenesis of influenza	2.61	2.65	CASP1, CCL2, EIF2AK2, ISG20, RIGI, STAT1, STAT2
ISGylation signaling pathway	2.61	1.41	DTX3L, EIF2AK2, HERC5, NFAT5, RIG1, STAT1, STAT2, TRIM25
JAK/STAT signaling	2.49	1.13	PIK3C3, PIK3R1, PTPN6, RAP1B, STAT1, STAT2, STAT4
Activation of IRF by cytosolic pattern recognition receptors	2.49	1.63	ADAR, IKBKB, RIG1, STAT1, STAT2, ZBP1
Interleukin-2 family signaling	2.40	2.24	PIK3R1, PTK2B, PTPN6, STAT1, STAT4
RIPK1-mediated regulated necrosis	2.22	2.00	CFLAR, HSP90AA1, MLKL, TNFSF10
Salvage pathways of pyrimidine ribonucleotides	2.17	1.89	CSNK1A1, EIF2AK2, NME1, UCK2, UCKL1, UPP1, UPRT

## Discussion

FM was previously characterized as a syndrome with widespread pain and localized tenderness^30^. Conceptually, the definition of FM has evolved over time and is perceived as a continuum representing an increased and heightened processing of pain within the nervous system^31^. In 2016, nociplastic pain was proposed as a mechanistic descriptor for FM and chronic pain. Nociplastic pain is defined as pain arising because of an increased sensitivity due to alterations in the peripheral and central nervous system^5^. In FM patients, nocipalstic pain can occur as a comorbidity with an inflammatory, immune, endocrine, genetic and psychosocial factors; all these phenotypes leading to a sensitization phenomenon characterized by a decrease in pain tolerance to afferent nociceptive stimuli^1,32,33^. Over the years, there has been increasing recognition that chronic pain conditions are heterogeneous with a degree of overlap of phenotypes^1,34^. However, to date, there is no clear explanation to account for this clinical heterogeneity. Our group reported a multiplex cytokine assay that could be used for achieving an objective diagnosis of FM patients^6^. The present study aimed to further define these patients via the identification of genomic markers or signatures to aid diagnosis and possibly lead to the development of mechanism based targeted therapy rather than symptom-based treatment. To this end, we utilized high throughput RNA-sequencing for whole transcriptome analysis in 94 patients with FM and 90 healthy control subjects (who had a negative cytokine assay result) using RNA from peripheral blood. The results of our analysis identified multiple subgroups within the cohort of FM patients with distinct non-overlapping gene signatures. Of note, subgroups of FM were identified with enough patients in two subgroups (FM1 and FM2) and combined FM3 and FM4 for detailed downstream analysis. The presence of multiple subgroups within FM patients reflects the inherent clinical heterogeneity associated with FM and chronic pain disorders which explains the diagnostic difficulty often encountered in a clinical setting. The two major subgroups displayed distinct transcriptional profiles indicating two different etiologies that are grouped together under the same general diagnosis of FM. Although we did not identify a specific cause of FM, identification of these subgroups will help develop additional novel diagnostic markers and therapeutics for these patients. The differences observed among the patients suggest that different treatment approaches will be required for patients with FM.

Our study identified subgroups of FM defined by transcriptional signatures. The first group, FM1, included individuals with a signature enriched for gene expression of extracellular matrix (ECM) associated with connective tissue disorders and down regulation of Rho GDP Dissociation Inhibitor (RhoGDI) signaling pathway. The second group, FM2, included individuals that showed a profound reduction in the expression of inflammatory mediators and increased expression of genes involved in the Coordinated Lysosomal Expression And Regulation (CLEAR) signaling pathway. The other two, FM3 and FM4 subgroups, while distinct from the FM1 and FM2, had two few subjects to clearly define the pathways involved. A combined analysis of FM groups 3 and 4 identified overexpression of interferon alpha/beta and JAK/STAT pathways and downregulation of the processing of capped intron containing pre mRNA pathway.

In the FM1 subgroup, among the biological processes regulated by the DEGs, include the processing of the ECM protein collagen, wound healing, fibrosis, and genes associated with cell cycle and DNA damage checkpoint regulation (Table 2). Genes associated with the RhoGDI signaling pathway were under-expressed. RhoGDI signaling pathway is the regulator of the Rho family of GTPases that are implicated in the formation of stress fibers and in pain perception through somatosensory neurons^35^. In this group of patients, deregulation of ECM and tissue homeostasis is likely mediated by fibrocytes. Fibrocytes are bone marrow-derived mesenchymal progenitor cells that directly contribute to tissue remodeling and fibrosis of tissues throughout the body by producing ECM proteins (collagen 1 and collagen III) and by secreting matrix metalloproteinases following injury, wound healing and during fibro-proliferative disorders in response to local tissue injury^36,37^. Fibrocytes traffic to sites of injury during the earliest phase of the innate immune response and exhibit both the inflammatory features of macrophages and the tissue remodeling properties of fibroblasts. They are also an important cellular source of inflammatory cytokines, chemokines, and growth factors that contribute to important autocrine and paracrine signals within the tissue microenvironment^38^. Fibrocytes are distinguished by the simultaneous expression of CD34 or CD45 and collagen^36,37^. Inhibition of Rho kinase increases resting tissue tension which regulates actomyosin contractility, the formation of stress fibers (actin-myosin filaments) and the maturation of focal adhesions^35,39^. Our results suggest that Rho-dependent remodeling of cell matrix is affected in the FM1 subgroup. At the local cellular level, matrix tension has been shown to influence a wide variety of cellular events including neurite growth and angiogenesis^40,41^. Thus, cell-mediated regulation of connective tissue tension may be important to protect blood vessels, sensory and autonomic nerves from prolonged tissue loads induced by various body positions such as sitting, standing, and sleeping positions. In vivo connective tissue tension may not only impact connective tissue homeostasis but also the vascular, nervous, and immune cell populations that reside within the connective tissue network as well as in adjacent organ-specific cell populations. The presence of cell cycle associated genes in the signature indicates persistent stimuli triggered by stress or chronic inflammation that leads to defects in DNA repair mechanisms prompting the activation of fibrocytes^42,43^.

In the FM2 subgroup, there was a significant immune dysregulation as reflected by the under expression of genes involved in phagosome formation, pyroptosis signaling, TREM1 signaling, neuro-inflammation, Th1 and Th2 pathways, crosstalk between dendritic cells and natural killer cells, toll-like receptor signaling and the inflammasome pathway, among others (Table 3). One of the top-ranked pathways that showed overexpression of genes includes the CLEAR signaling pathway. CLEAR pathway is a cellular program that regulates lysosomal biogenesis and participates in macromolecule clearance^44^. CLEAR network is activated by lysosomal storage. The transcription factor EB (TFEB) is a master regulator of lysosomal function^44,45^. TFEB promotes the expression of genes involved in lysosomal biogenesis, such as the mannose 6-phospate receptors, which transport newly synthesized lysosomal enzymes from Golgi to lysosomes. The activity of TFEB is regulated by multiple kinases, in particular the mechanistic target of rapamycin complex 1 (mTORC1)^46–48^. When phosphorylated, TFEB is retained in the cytoplasm and inhibited. Several stress signals including nutrient deprivation, proteotoxicity, and lysosomal damage, which have been reported to promote TFEB dephosphorylation, nuclear translocation and activation, leading to an increase in the number and activity of lysosomes^49^. mTORC1 is activated by nutrients and growth factors, and conversely is inhibited by starvation^50^. The activation or inactivation of mTORC1 in response to nutrient availability occurs on the lysosome and is regulated by several lysosomal membrane-associated proteins^51,52^. Thus, the lysosome not only functions as a scaffolding organelle but also participates in the nutrient sensing process. The regulation of mTORC1 signaling by the lysosome also occurs through a transcriptional mechanism mediated by TFEB, which is activated in response to lysosomal stress. TFEB direct target genes were identified by combining ChIP-seq, TFEB overexpression, promoter analysis and co-expression meta-analysis^53^. These genes encode for proteins that can be grouped into several distinct categories, including lysosomal hydrolases and accessory proteins, lysosomal membrane proteins, subunits of the proton pump, proteins participating in autophagy and non-lysosomal proteins involved in lysosomal biogenesis^53^. Our data show differential expression of genes encoding lysosomal hydrolases and accessory proteins ASAH1, GAA, GNS, IFI30, PSAP; and genes involved in lysosomal acidification ATP6V0B, ATP6V0C, ATP6V0D1, ATP6V1B2 (Table 3) suggesting dysregulation of lysosomal homeostasis in FM2 patients. TFEB also promotes the formation of autophagosomes and their fusion with lysosomes through the upregulation of several key autophagy and lysosomal genes, a process that is initiated by nutrient starvation and executed by the inhibition of extracellular signal regulated kinase 2 (ERK2)-mediated phosphorylation of TFEB at Ser142^48^. Our results show differential expression of the autophagy gene GABA type A receptor–associated protein (GABARAP) (Table 3). GABARAP is a ubiquitin-like modifier that plays a role in intracellular transport of GABA(A) receptors and its interaction with the cytoskeleton. It is involved in autophagy while the microtubule-associated protein 1A/1B-light chain 3 (LC3) is involved in elongation of the phagophore membrane. The GABARAP subfamily is essential for a later stage in autophagosome maturation^54^. Through its interaction with the reticulophagy receptor TEX264, GABARAP participates in the remodeling of subdomains of the endoplasmic reticulum into autophagosomes upon nutrient stress, which then fuse with lysosomes for endoplasmic reticulum turnover^55^. Other TFEB direct targets are genes belonging to distinct families of pattern recognition molecules including membrane-anchored Toll-like receptors (TLRs), which are involved in the innate immune detection of danger signals and microbial motifs^56^ and the insulin signaling pathway^53^. Taken together, these results indicate defects in vesicle transport and lysosomal homeostasis in FM2 patients.

In the FM3 and FM4 patients, we identified pathways related to acute inflammatory associated with the Th1 responsive processes with overexpression of interferon pathway, JAK/STAT pathway, IL2, pyroptosis, cell death receptor and necroptosis pathways. Strong down regulation of processing of pre-mRNA pathway indicates global dysregulation of the transcription machinery (Table 4).

There were many limitations in this study inherent to conducting research involving live human subjects and a disease where biology is poorly understood. This study was biased because only individuals who tested positive for the cytokine assay (FM/a) were included. Although this test was developed and validated by our group, it is not widely used or validated by an outside group. Since these criteria were used for patient selection, the study was inherently biased. However, all analyses were performed using the clinical diagnostic criteria for FM recommended by the American College of Rheumatology. We also included only patients with FM rather than patients with chronic pain. In that regard the study is biased towards a subset of patients with FM and the results may not apply to all patients with chronic pain disorder. Future studies are necessary to gain insight into the biological problems in people with chronic pain.

In conclusion, the whole transcriptome analysis of FM patients identified novel gene expression signatures. To our knowledge, this is the first study to report genetic heterogeneity within FM patients. The two major groups of FM patients reported here have defects in the tissue homeostasis associated with ECM and the lysosomal biogenesis pathway. We provide possible mechanisms of FM pathogenesis that need to be further validated to gain precise understanding of the biology of FM and develop novel treatment approaches.

### Supplementary Information

Supplementary Figure 1.

Supplementary Figure 2.

Supplementary Legends.

Supplementary Table 1.

## Data Availability

Raw data have been submitted to NCBI and GSE221921 provides access to all data (https://www.ncbi.nlm.nih.gov/gds/?term=GSE221921). List of differentially expressed genes that are used in the analysis to support the figures are provided in supplementary table 1.
