# Supplementary figures and images for: Identification of unique genomic signatures in patients with fibromyalgia and chronic pain
Source: Sci Rep. 2024 Feb 17;14:3949. doi: 10.1038/s41598-024-53874-8 (PMC10873305; doi:10.1038/s41598-024-53874-8)

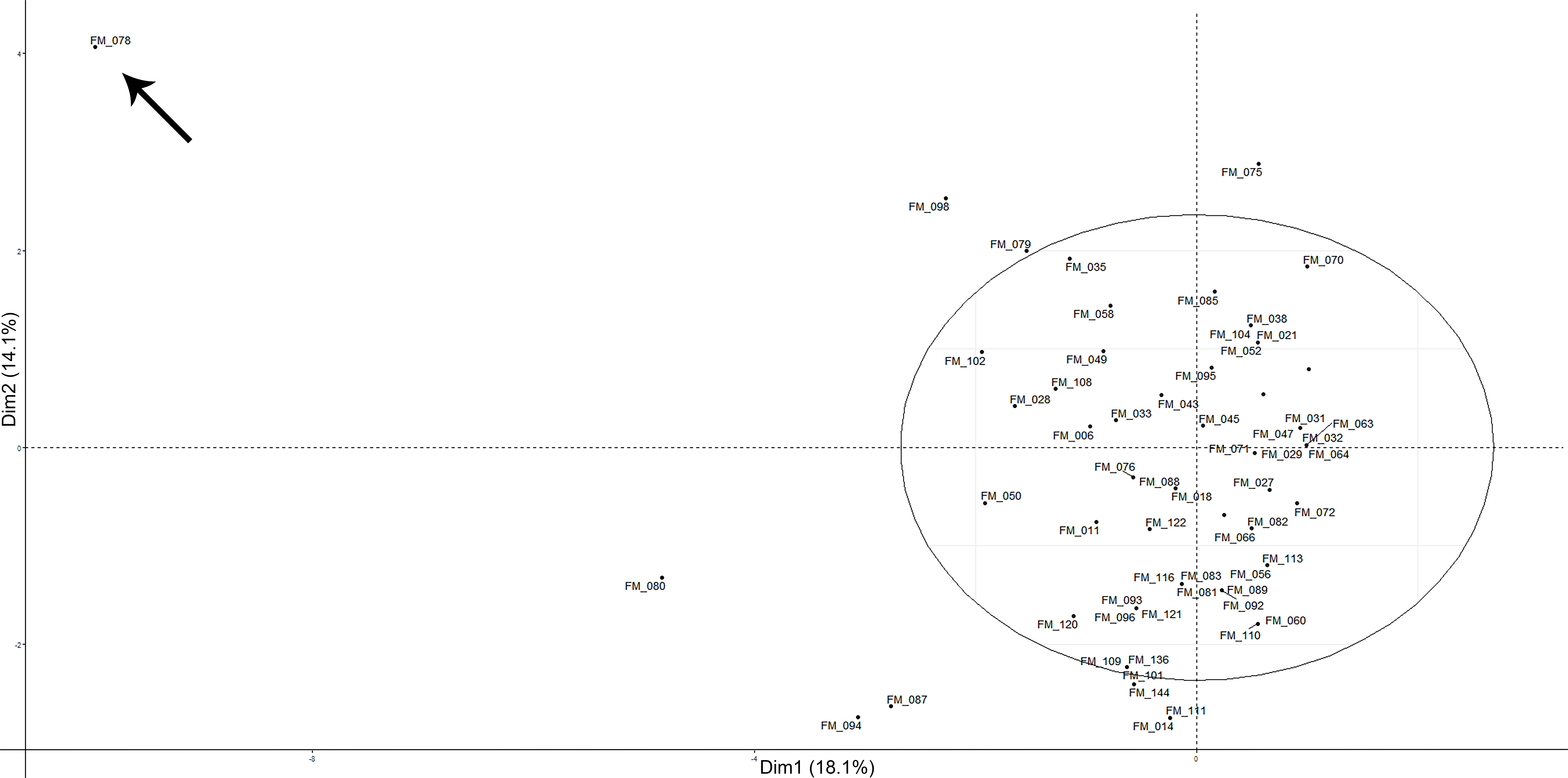

Supplement: Supplementary file 1 — Supplementary Figure 1. [file 41598_2024_53874_MOESM1_ESM.tif]

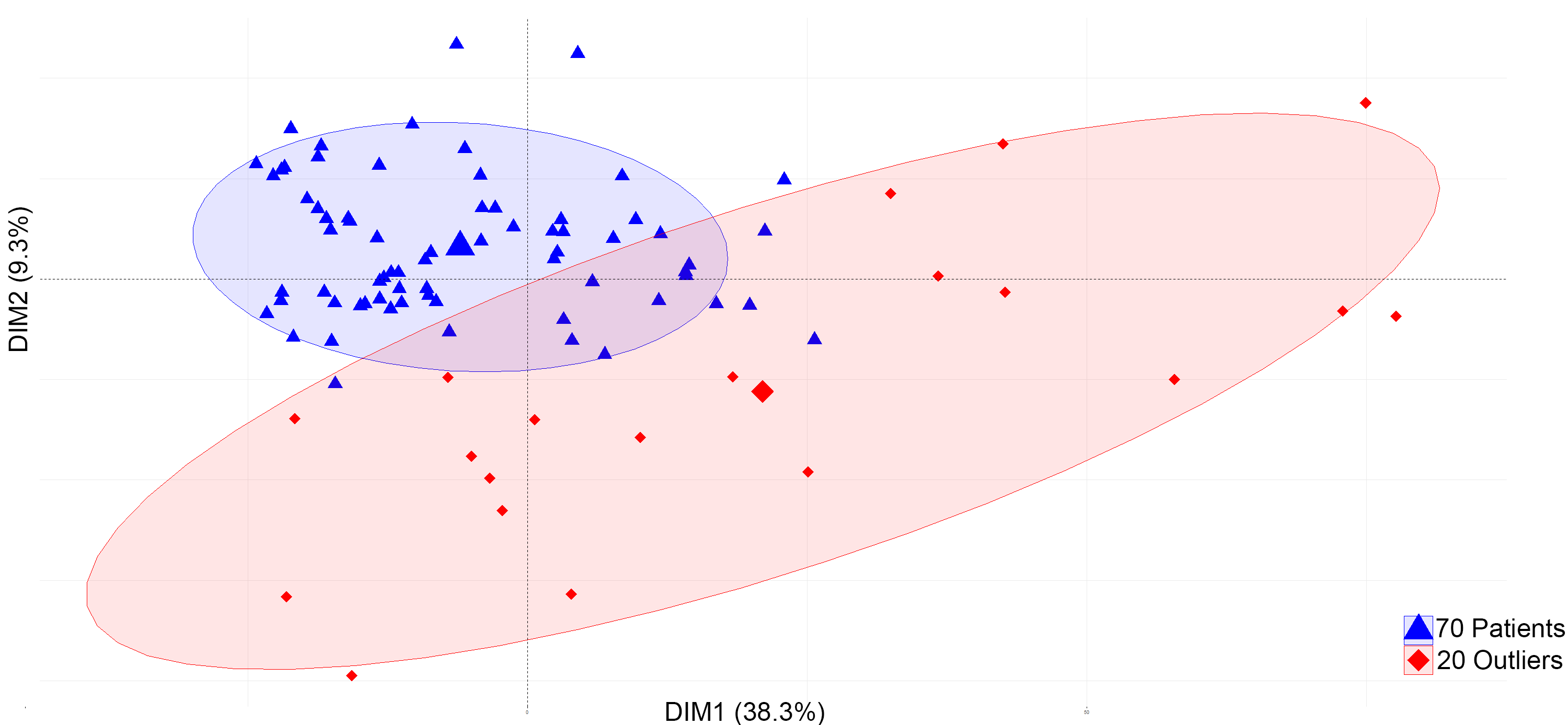

Supplement: Supplementary file 2 — Supplementary Figure 2. [file 41598_2024_53874_MOESM2_ESM.tif]
