# Supplementary material for: Identification of unique genomic signatures in patients with fibromyalgia and chronic pain
Source: Sci Rep. 2024 Feb 17;14:3949. doi: 10.1038/s41598-024-53874-8 (PMC10873305; doi:10.1038/s41598-024-53874-8)
Supplement: Supplementary file 3 — Supplementary Legends. [file 41598_2024_53874_MOESM3_ESM.docx]

Supplementary material legends

Supplementary Figure 1. PCA of the symptoms in FM patients

Supplementary Figure 2.PCA of the control patients using the DEGs

Supplementary Table 1.List of genes related to FM subgroups
